# The Chinese version of Selection, Optimization, and Compensation Questionnaire: psychometric properties among adolescent students

**DOI:** 10.3389/fpsyg.2025.1453882

**Published:** 2025-02-05

**Authors:** Mengjun Zhu, Xing’an Yao, Abu Talib Mansor

**Affiliations:** ^1^Wellbeing Research Centre, Faculty of Social Sciences and Liberal Arts, UCSI University, Kuala Lumpur, Malaysia; ^2^School of Marxism, Nanjing Institute of Technology, Nanjing, China

**Keywords:** intentional self-regulation, regulation strategies, validation, reliability, adolescent

## Abstract

**Objective:**

This study established the reliability and validity of the Selection, Optimization, and Compensation Questionnaire in a sample of senior secondary school students in China.

**Methods:**

The data for this study were sourced from 1,080 students from multiple senior secondary schools using the SOC Questionnaire and the Learning Engagement Scale. This study conducted Exploratory Factor Analysis and Confirmatory Factor Analysis. The factor structure analysis of the psychometric properties of SOC Questionnaire were examined on reliability, convergent validity, concurrent criterion validity, and incremental validity.

**Results:**

The EFA results suggested that a three-factor solution was most appropriate for the SOC Questionnaire. The three-factor CFA model of this study calculated correlations different from what was published with an American sample of adolescents by the questionnaire developers. The reliability coefficients (Cronbach’s *α*, McDonald’s *ω*), composite reliability (rho_c), and reliability coefficient (rho_a), convergent and discriminant validity were good. Concurrent criterion validity, and incremental validity were demonstrated by the SOC Questionnaire and the LES.

**Conclusion:**

The 17-item, 3-factor SOC Questionnaire demonstrated strong reliability and validity, thus offering a new multidimensional of the SOC Questionnaire to evaluate intentional self-regulation among adolescents in schools.

## Introduction

Research into the mechanisms underlying intentional self-regulation became one of the central topics in developmental psychology (Geldhof et al., 2015a,b; Gestsdottir and Lerner, 2008). ISR coincides with how people can control their thoughts, emotions, and behavior to achieve goals. It is an essential feature across all of life. An essential instrument for measuring ISR is the Selection, Optimization, and Compensation Questionnaire, based upon the Baltes and Baltes (1990) theoretical framework. The central strategies of the SOC model are called selection, optimization, and compensation. Each refers to setting and prioritizing goals, allocating and refining resources for goal attainment, and using alternative strategies in the maintenance process, respectively, (Freund and Baltes, 2002). The SOC Questionnaire has been widely applied across different age groups and cultural contexts, showing all the diversity in self-regulation it can tap into in diverse populations (Geldhof et al., 2015a,b; Okabayashi, 2014; Viglund et al., 2013). Research into SOC strategies recently focused on themes such as work, aging, and personal growth, emphasizing enhancing adaptability and wellbeing among people (Moghimi et al., 2021; Zając-Lamparska, 2021). Therefore, SOC interventions depend on constructing proper measurement instruments that must be tailored according to different cultural contexts (Okabayashi, 2014; Viglund et al., 2013). Even though multidimensional self-report questionnaires about the SOC exist for adults, until now, corresponding questionnaires for adolescent populations were still theoretically incoherent and culturally inappropriate (Meng et al., 2021; Millová and Malatincová, 2021; Zając-Lamparska, 2021). Therefore, it is quintessential to adapt and validate the SOC Questionnaire in Chinese for further understanding of ISR in a Chinese context.

Although the SOC Questionnaire has been used to understand ISR, validation studies in different cultural contexts, especially among adolescents, have been very few (Geldhof et al., 2015a,b; Meng et al., 2021; Millová and Malatincová, 2021; Viglund et al., 2013). Existing literature shows that while the SOC model has had a place within Western cultures, it is still significantly underexplored in its application to non-Western settings—a blind spot in the literature (Gestsdottir et al., 2015). Cultural differences may mean variations in perceiving and reporting ISR by individuals and, hence, biases in measurement. For example, varying social norms and values in adolescents may, at times lead to different replies to the SOC Questionnaire items, which may hamper the tool’s validity (Viglund et al., 2013). Translation and adaptation of the SOC Questionnaire into various languages should thus be validated with proper scrutiny to prove its reliability and validity across cultural contexts (Meng et al., 2021). Such studies on comprehensive cross-cultural validation will be significant in establishing the efficiency of the SOC Questionnaire in global adolescent populations, thus furthering the study of self-regulation. It will offer invaluable insights into cultural differences in teenage development, hence promoting this research area (Geldhof et al., 2015a,b).

Some studies of the use of the SOC Questionnaire across different cultures revealed that while the Czech and Polish versions of the SOC Questionnaire had sufficient reliability and validity, further refinement was needed to better capture age-specific and cultural differences (Millová and Malatincová, 2021; Zając-Lamparska, 2021). In the classroom environment, SOC strategies are positively related to the pupils’ academic performance and self-efficacy beliefs; thus, optimization strategies would be an imperative resource within the school and college environment (Moghimi et al., 2021). Later scholars have evidenced immense variation in the sources of SOC strategies used within age ranges across different individuals. The amounts of strategy use increased at midlife, whose impact was substantial in sort of wellbeing and impulsivity during that curvature age period (Zając-Lamparska, 2021). The present findings further underscore the importance of continued work in confirming the applicability of the SOC Questionnaire with a culturally diverse adolescent population and extending this work to other cultural settings (Meng et al., 2021).

Although intentional self-regulation is a growing concept in China, especially among adolescents, very few studies have been carried out on developing and validating measuring tools such as the SOC Questionnaire. A search across journals for publications within the last 10 years relating to Chinese adolescents resulted in no findings regarding developing and validating ISR questionnaires for adolescents. Although both the Academic and Social Selection, Optimization, and Compensation (SOC) Questionnaire by Millová and Malatincová (2021), or the Polish SOC Questionnaire proposed by Zając-Lamparska (2021), can be helpful during research with adolescents for a general understanding of ISR, essential limitations have been noticed. Although, in general, the SOC model is purportedly a multidimensional construct that consists of several specific subcomponents, the measurement tool—existing SOC Questionnaires measuring ISR—may have different dimensional structures at different age developmental stages, probably because of the applicability problems of these measurement tools (Gestsdottir et al., 2015). Meanwhile, ISR heterogeneity also makes it difficult for any existing SOC Questionnaires to capture all its multidimensional structure and complexity (Moghimi et al., 2021). These questionnaires create problems for the measurement of self-regulation strategies in adolescents in non-Western cultural contexts and significantly restrict the possibility of investigating the complex structure of ISR (Zając-Lamparska, 2021). Taking as a point of departure the aspiration to deepen knowledge about ISR in adolescent populations and increase the chances of carrying out adequate ISR studies among culturally diverse adolescent populations, this paper aims to translate and validate a multidimensional adolescent self-report SOC Questionnaire.

Translation and adaptation of the SOC Questionnaire will be done, which was initially designed to assess the level of intentional self-regulation expended by an individual in effectively managing their goals and resources. There are 18 items and three dimensions within the questionnaire: Selection, Optimization, and Compensation. As stated by Freund and Baltes (1998), each of the dimensions of the SOC Questionnaire captures critical aspects of ISR that enable a comprehensive understanding of how individuals manage their development and wellbeing. The development and validation of this SOC Questionnaire have been checked in various cultural contexts, such as the Czech Republic, Poland, and Japan. As research has been able to show, the questionnaires measure intended constructs but underline the further adaptation needed to catch cultural and age-specific differences (Millová and Malatincová, 2021; Okabayashi, 2014; Zając-Lamparska, 2021). Previous studies have shown that using the SOC Questionnaire among first-year university students was positively correlated with wellbeing and academic performance; therefore, this instrument is applicable within the educational environment (Moghimi et al., 2021). Considering the foregoing, this research is currently being done for the translation and cultural adaptation of this tool to Chinese adolescents. This includes not only the linguistic translation of instruments but also the modulation of specific cultural values and developmental stages to be more representative of Chinese students. In this regard, it will help tighten gaps in cross-cultural ISR research, furthering the development of effective interventions adjusted for different groups of adolescents (Meng et al., 2021).

Preliminary validation among adolescents was conducted by Gestsdottir et al. (2015) in Canada, Germany, Iceland, and the United States, with further validation needed using more extensive and more diverse adolescent samples. The present study aims to establish the reliability and validity of a translated SOC Questionnaire on Chinese teenage students. Its objectives are to assess the psychometric properties of the translated SOC Questionnaire. First, the translated SOC Questionnaire structure will be explored and validated for its cultural adaptation with theoretical consistency. Second, the reliability and internal consistency of the questionnaire would be estimated by Cronbach’s alpha and McDonald’s omega (McDonald, 1999). The convergent and discriminant validity regarding the SOC Questionnaire will be estimated using standard indicators of related constructs, such as the Learning Engagement Scale (Fang et al., 2008). The concurrent criterion and incremental validity of the SOC subscales concerning engagement in learning will be checked by correlation and hierarchical regression analyses.

## Methods

### Participants

This study employed a multi-stage stratified cluster sampling method to recruit participants from grades 10 to 12 across eight middle schools in Central China. A total of 1,220 questionnaires were distributed through the online platform “Questionnaire Star” across 12 classes, ensuring comprehensive and efficient data collection. To achieve randomness and representativeness, a three-stage sampling process was conducted. In the first stage, cluster random sampling was used to select four regions from Central China, ensuring geographic and socio-economic diversity. In the second stage, two schools were randomly selected from each region using simple random sampling. In the third stage, three grade levels (10th, 11th, and 12th) were chosen from each school using stratified sampling, followed by cluster random sampling to select one class per grade.

After excluding invalid questionnaires due to errors in control questions, patterned responses, or incomplete data, the final sample comprised 1,080 participants, with an effective recovery rate of 88.52%. The sample included 558 males (51.67%) and 522 females (48.33%), with 362 in 10th grade (33.52%), 368 in 11th grade (34.07%), and 350 in 12th grade (32.41%). Among the participants, 566 were urban students (52.41%) and 514 were rural students (47.59%), aged between 15 and 18 years, with an average age of 16.56 years (*SD* = 0.90). According to the sample size selection standards proposed by Comrey and Lee (1992), a sample size exceeding 500 is considered very good, ensuring sufficient statistical power in factor analysis to validate the questionnaire’s factor structure. The sample size used in this study meets these requirements.

### Procedure

Data collection was conducted using the online survey tool “Wenjuanxing”,^1^ which facilitated questionnaire management and ensured that participants could only submit the questionnaire after completing all items, minimizing the likelihood of missing responses. Before starting the survey, detailed information about the study, including its purpose and participants’ rights, was provided. Ethical procedures were strictly followed, informing participants of the voluntary nature of their participation and the confidentiality of their responses. All respondents gave informed consent and could withdraw from the study at any time. To enhance the validity of responses, several control questions were included to detect and exclude patterned or inconsistent answers. The data were then randomly divided into two equal samples using SPSS’s random selection algorithm. Exploratory Factor Analysis (EFA, *N* = 540) was conducted to explore the underlying factor structure, followed by Confirmatory Factor Analysis (CFA, *N* = 540) to confirm the factor structure, thereby supporting the robustness of the study results.

### Measures

The demographic questionnaire collected information on age, gender, grade level, number of siblings, place of residence, and family economic status (see Table 1).

**Table 1:** Demographic profile of respondents (*N* = 1,080).

Demographic	Level	Frequency	Percentage (%)
Age	15	138	12.78
	16	360	33.33
	17	416	38.52
	18	166	15.37
Gender	Boy	558	51.67
	Girl	522	48.33
Grade	10th	362	33.52
	11th	368	34.07
	12th	350	32.41
Number of siblings	0	744	68.89
	1	308	28.52
	2	28	2.59
Household location	Urban	566	52.41
	Rural	514	47.59
Family economic status (monthly income)	Below 2,000	6	0.56
	2,001–5,000	22	2.04
	5,001–8,000	278	25.74
	8,001–10,000	560	51.85
	Above 10,000	214	19.81

### Selection, Optimization, and Compensation (SOC) Questionnaire

The brief version of the Selection, Optimization, and Compensation (SOC) Questionnaire was used to measure the ability of intentional self-regulation (Freund and Baltes, 2002). This three-dimensional questionnaire measures Selection (S), Optimization (O), and Compensation (C) with 18 items, 6 items per dimension. All items on the SOC Questionnaire are positively phrased, such as “I concentrate all my energy on few things.” (Selection), “I think about exactly how I can best realize my plans.” (Optimization), and “When things aren’t going so well, I accept help from others.” (Compensation). Students responded to each item using a 5-point Likert scale. While McDonald’s omega values for the full scale were not provided, the Cronbach’s alpha values for the three subscales in the American sample were reported as 0.75 for Selection, 0.70 for Optimization, and 0.67 for Compensation.

To ensure the accuracy and adaptability of the SOC Questionnaire in cross-cultural research, this study employed a comprehensive multi-stage translation method incorporating best practices to ensure the equivalence and reliability of the questionnaire content across different cultural contexts. Initially, two bilingual researchers independently translated the SOC Questionnaire from English into Chinese. The translated scale was then proofread by two certified translators. Next, a bilingual psychology professor reviewed the translated content for fluency and cultural appropriateness. Subsequently, a native English-speaking bilingual individual conducted a blind back-translation without referring to the original questionnaire, which was then compared to the original. Finally, a pilot test was conducted with a convenience sample of 15 high school students in the target culture to analyze item comprehension and make necessary adjustments, ensuring high reliability and readability in the new cultural context. Participants reported no difficulties in understanding and responding to the scale. Additionally, two statements originally scored in a binary manner were converted to a 5-point Likert scale, requiring respondents to quantify their agreement with the SOC Questionnaire statements (1 indicating “strongly disagree” and 5 indicating “strongly agree”). The Likert-type response format may be an equally valid and potentially more practical alternative to the binary forced-choice response format (Geldhof et al., 2015a,b). Scores on the SOC Questionnaire represent the level of intentional self-regulation. In this study, the Cronbach’s *α* and McDonald’s *ω* coefficients for the scale were 0.93 and 0.93, respectively.

### Learning engagement scale (LES)

The 17-item Learning Engagement Scale (Schaufeli et al., 2002) was used to assess the level of learning engagement. This scale has been revised and validated among Chinese adolescents (Fang et al., 2008). The 17 items are distributed across three dimensions: Vigor (e.g., “I am enthusiastic about learning in the morning”), Dedication (e.g., “I find learning challenging”), and Absorption (e.g., “I often forget everything around me while studying”). The “Vigor” dimension includes 6 items (1–6), the “Dedication” dimension includes 5 items (7–11), and the “Absorption” dimension includes 6 items (12–17). Responses were made on a 7-point Likert scale ranging from 1 (never) to 5 (always). The Chinese version of the LES demonstrated satisfactory reliability indicators: 0.86 for Vigor, 0.91 for Dedication, and 0.90 for Absorption. Each item described students’ learning engagement, with higher scores indicating higher levels of engagement. In this study, the Cronbach’s *α* and McDonald’s *ω* coefficients for the scale were 0.94 and 0.94, respectively.

### Data analyses

Data analysis was performed using SPSS 27.0 and Smart PLS v.4.1.0.0 software. Data were collected through the “Questionnaire Star” online platform to avoid any missing data. Initially, data screening was conducted to check for suspicious response patterns or outliers. Outliers were detected using Cook’s distance, with values greater than 1.0 potentially indicating anomalies (Field, 2018). Common method bias or common method variance was also examined. Harman’s single-factor test was used, and if the first factor explained less than the critical threshold of 50% variance, common method bias was considered absent (Yüksel, 2017). To verify the normality of the observed data, skewness and kurtosis values were calculated, with values within ±2 considered acceptable (Field, 2018). Additionally, multicollinearity issues were checked using the variance inflation factor (VIF) (1 < VIF < 5) (Marcoulides and Raykov, 2019).

Subsequently, the first sample (*N* = 540) was used for Exploratory Factor Analysis (EFA) to determine the factor structure of the SOC Questionnaire. One-factor, two-factor, and three-factor models were tested (Preacher et al., 2013). To assess the adequacy of the sample and the suitability of factor analysis, three criteria were used: Kaiser-Meyer-Olkin (KMO > 0.60) (Cerny and Kaiser, 1977) and Bartlett’s test of sphericity (*p* < 0.05) (Bartlett, 1950), communalities (>0.50; Hair et al., 2010), and factor loadings (>0.40; Stevens, 2009). Principal Component Analysis was used with Varimax and orthogonal rotation to optimize sub-domains’ loadings on extracted factors. A minimum factor loading threshold was set at 0.50; items loading below 0.50 were removed (Hair et al., 2010).

After EFA, using the second sample, CFA was conducted to validate the three-factor model with the help of the second sample (*N* = 540). For this, CB-SEM was conducted using Smart PLS v.4.1.0.0. In this measurement of model fit, which is primarily based on Boomsma (2000), several fit indices were used, including *χ*^2^, RMSEA, GFI, CFI, and the ratio *χ*^2^/df. The *χ*^2^ test was, however, not considered since this is greatly influenced by the sample size, especially when it is more than 200, usually giving a *p*-value of 0.000 (Jöreskog and Sörbom, 1993). This made the researchers ignore the *χ*^2^ test. Instead, the following robust indicators were adopted: Ratio *χ*^2^/df, GFI, CFI, and RMSEA, because according to Kline (2016), these indicators evaluate how well the *a priori* or hypothesized model represents the sample data. The model fit indices, according to Schumacker and Lomax (2012), are Ratio *χ*^2^/df < 5.00 and CFI and GFI ≥ 0.90 and RMSEA ≤ 0.08.

Several reliability measures were computed to evaluate further the reliability and validity of the SOC Questionnaire, including Cronbach’s alpha, McDonald’s *ω*, Normed MSA, Cronbach’s alpha if item deleted, composite reliability (rho_c), reliability coefficient (rho_a), and Average Variance Extracted (AVE). Confidence intervals for each reliability coefficient have been generated using 5,000 bias-corrected, accelerated BCa bootstrap samples (Hair et al., 2022). It is strongly advised to use the BCa bootstrap method developed by Efron (1987) for its robust function of adjusting bias and skewness in the bootstrap distribution. Meanwhile, if the zero does not fall within a 95% CI, the correlation coefficient would be statistically significantly different from zero. Reliability and internal consistency for the SOC Questionnaire were computed by reporting Cronbach’s alpha (>0.70) and McDonald’s composite reliability (MCR, >0.60; McDonald, 1999). Individual variable appropriateness was examined using Normed MSA (>0.50), with values closer to 1 indicating a more excellent suitability for inclusion into factor analysis. Cronbach’s alpha if item deleted was to test the changes in Cronbach’s alpha after deleting a specific item or items to identify which items may affect reliability. While the rho_c and rho_a respectively, attain values typically larger than Cronbach’s alpha, acceptable rho_c and rho_a should be more significant than 0.70 (Dijkstra and Henseler, 2015). The AVE was calculated as more significant than 0.50 for its item loadings to report the discriminant validity of the SOC Questionnaire. For the Cornell-Larcker criterion, the requirement is that the square roots of each construct’s AVE be more significant than its correlation with other constructs. Convergent validity was estimated by checking the correlation of the SOC Questionnaire with the Learning Engagement Scale. Systematically, using Smart PLS version v.4.1.0.0 by Ringle et al. (2014), internal consistency reliability and convergent and discriminant validity for the subscales were assessed with 5,000 bootstrap samples. Finally, the authors checked for concurrent and incremental validity using correlation and hierarchical regression analyses in SPSS 27.0. Here, scores on the SOC Questionnaire predicted Learning Engagement Scale scores, where Δ*R*^2^ served as the criterion measure of prediction.

## Results

### Item analysis

Descriptive statistics for the items are presented in Table 2. The mean scores for the Selection dimension ranged from 3.00 to 3.17, for the Optimization dimension from 3.01 to 3.05, and for the Compensation dimension from 2.96 to 3.07. Skewness and kurtosis values were within the acceptable range of −2 to 2, supporting the use of maximum likelihood estimation for Exploratory Factor Analysis (EFA). These results indicate that the data are suitable for factor analysis. VIF values ranged from 1 to 5, indicating no multicollinearity issues. Additionally, Corrected Item-Total Correlation (CITC) values ranged from 0.35 to 0.74, indicating moderate to strong correlations between all items and the total score.

**Table 2:** Descriptive statistics of items.

Label	Mean	SD	Min	Max	Skewness	Kurtosis	VIF	CITC
S1	3.17	1.03	1	5	−0.09	−0.67	1.90	0.58
S2	3.08	1.06	1	5	−0.05	−0.63	2.08	0.59
S3	3.00	1.05	1	5	−0.07	−0.62	2.03	0.56
S4	3.00	1.06	1	5	−0.08	−0.55	2.15	0.58
S5	3.15	0.99	1	5	−0.18	−0.31	2.10	0.57
S6	3.25	0.96	1	5	−0.21	−0.42	1.78	0.55
O1	3.04	1.10	1	5	0.02	−0.71	2.78	0.70
O2	3.04	1.10	1	5	0.07	−0.72	2.84	0.67
O3	3.02	1.11	1	5	0.05	−0.69	2.63	0.68
O4	3.01	1.10	1	5	0.04	−0.68	2.76	0.69
O5	3.05	1.06	1	5	0.01	−0.64	1.22	0.35
O6	3.01	1.07	1	5	0.12	−0.66	3.84	0.71
C1	3.03	0.98	1	5	0.09	−0.51	1.82	0.63
C2	2.97	0.99	1	5	0.08	−0.35	3.64	0.74
C3	2.96	1.14	1	5	0.18	−0.75	3.15	0.72
C4	3.05	1.16	1	5	0.09	−0.79	2.71	0.68
C5	3.07	1.18	1	5	0.04	−0.84	3.07	0.70
C6	3.00	1.16	1	5	0.13	−0.82	3.30	0.73

S, Selection; O, Optimization; C, Compensation; CITC, Corrected Item total correlation.

### Construct validity

In this study, the Kaiser-Meyer-Olkin (KMO) value for the SOC Questionnaire was 0.95, and Bartlett’s test of sphericity was significant (*χ*^2^(153) = 6,357.49, *p* < 0.001), supporting the data’s suitability for Exploratory Factor Analysis. EFA was conducted using Principal Component Analysis and Varimax rotation, and factors with eigenvalues greater than 1 were retained. Three factors were extracted from the SOC Questionnaire, with the scree plot further supporting the three-factor model, consistent with the original structure. Most items had significant factor loadings (>0.50) on the Selection, Optimization, and Compensation factors, except for item 10. Item 10 had a factor loading of 0.44 and a communality of 0.29, which did not meet the acceptable thresholds (factor loading >0.50, communality >0.30). Therefore, to ensure the robustness and clarity of the factor structure, item 10 (“When I start something that is important to me but has little chance at success, I usually stop trying.”) was removed from further analysis. The remaining 17 items explained 24.65, 21.58, and 21.20% of the variance for the three factors, respectively, with a total variance explained of 67.43%. Detailed data for the factor analysis are shown in Table 3.

**Table 3:** The 18-item SOC’s rotated factor matrix of 3-factor EFA with eigenvalues and goodness-of-fit indices (*N* = 540).

Construct	Items	Factor loading			Commonalities
		Factor1 (S)	Factor2 (O)	Factor3 (C)	
Selection	Item 1		0.72		0.60
	Item 2		0.76		0.65
	Item 11		0.75		0.63
	Item 12		0.77		0.66
	Item 13		0.78		0.66
	Item 18		0.71		0.57
Optimization	Item 3			0.76	0.73
	Item 6			0.78	0.75
	Item 7			0.77	0.72
	Item 8			0.76	0.72
	Item 10			0.44	0.29
	Item 14			0.84	0.82
Compensation	Item 4	0.59			0.50
	Item 5	0.82			0.79
	Item 9	0.80			0.76
	Item 15	0.81			0.74
	Item 16	0.83			0.78
	Item 17	0.81			0.78

Principal component extraction and Varimax rotation; S, Selection; O, Optimization; C, Compensation.

**Figure 1:**
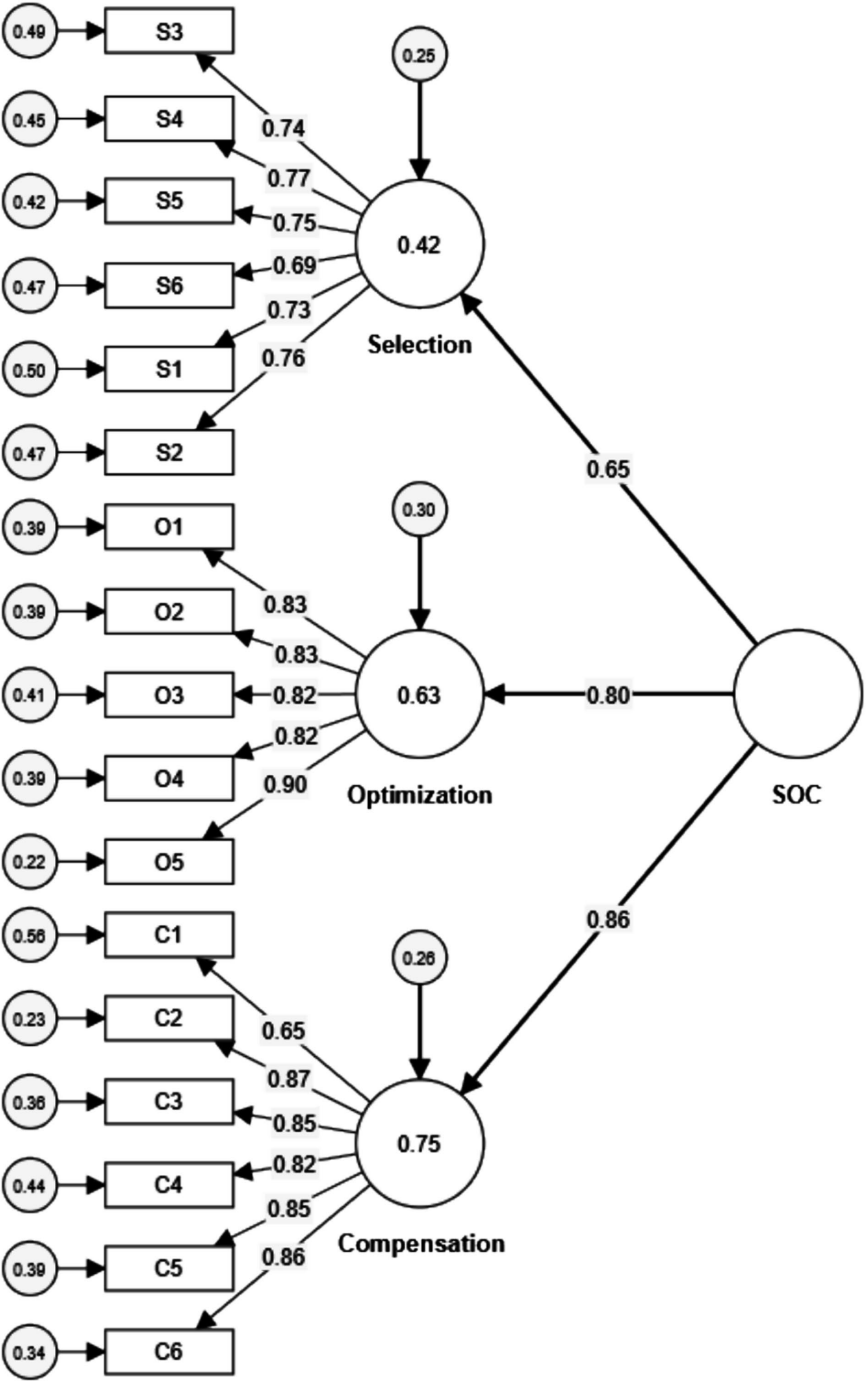
Confirmatory factorial analyses for the SOC Questionnaire.

Following EFA, Confirmatory Factor Analysis (CFA) was conducted using another sample (*N* = 540) with maximum likelihood estimation (see in Figure 1). The initial model fit indices indicated a good fit (*χ*^2^(116) = 182.11, *χ*^2^/df = 1.57, GFI = 0.96, CFI = 0.99, RMSEA = 0.03) (Table 4). Table 3 summarizes the descriptive statistics and factor loadings for the items within each dimension of the SOC Questionnaire. Factor loadings for the Selection factor ranged from 0.69 to 0.77, for the Optimization factor from 0.82 to 0.90, and for the Compensation factor from 0.65 to 0.87. All items loaded significantly on their respective dimensions, and all 17 factor loadings were statistically significant (Table 3). The overall Cronbach’s *α* and McDonald’s *ω* for the scale were 0.94. The internal consistency reliability, convergent validity, and discriminant validity of the subscales were assessed using 5,000 bootstrap samples with Smart PLS v.4.1.0.0. Subsequent hierarchical regression in SPSS was used to test the predictive/incremental validity.

**Table 4:** CFA model fit indexes and the results of model fit testing.

Model fit index	Benchmark for model fit	Model testing result	Fit interpretation
*χ*^2^/df	<5.00	*χ*^2^/df = 1.57	Acceptable
GFI	≥0.90	GFI = 0.96	Acceptable
CFI	≥0.90	CFI = 0.99	Acceptable
RMSEA	≤0.08	RMSEA = 0.03	Acceptable

Average Variance Extracted (AVE) was used to assess the convergent validity of the subscales. The minimum acceptable AVE value is 0.50, indicating that a factor (e.g., SOC) should explain at least 50% of the variance among the items (Hair et al., 2022). As shown in Table 6, the AVE for the SOC subscales ranged from 0.55 to 0.70, meeting the recommended standard of 0.50. Additionally, convergent validity requires that the square root of each factor’s AVE must be greater than the factor’s inter-correlations (Fornell and Larcker, 1981). As shown in Table 4, the square root of the AVE was greater than the inter-construct correlations, indicating good convergent validity for the three subscales.

The Heterotrait-Monotrait Ratio (HTMT) should be less than 0.85, indicating no issues with discriminant validity (Henseler et al., 2014). The HTMT between Selection and Optimization was 0.52, between Selection and Compensation was 0.57, and between Optimization and Compensation was 0.71, all below 0.85, indicating good discriminant validity for the SOC Questionnaire.

### Concurrent criterion validity

Correlation analyses were conducted between the three subscales of the LES and the three subscales of the SOC using SPSS 27.0. Table 5 presents the correlation matrix between the three factors of the SOC (Selection, Optimization, and Compensation) and the three factors of the LES (Vigor, Dedication, and Absorption). There were significant moderate positive correlations between the SOC subscales and the learning engagement factors, ranging from 0.25 to 0.44.

**Table 5:** The SOC’s subscale correlations and HTMT indices for validity analysis.

Subscale	Inter-factor correlation (HTMT index)			Sqrt. (AVE)
	Selection [95% CI]	Optimization [95% CI]	Compensation [95% CI]	
Selection	—	(0.52)^a^	(0.57)^a^	0.74 (0.55)
Optimization	0.52**	—	(0.71)^a^	0.84 (0.70)
Compensation	0.56**	0.69**	—	0.82 (0.67)
Vigor	0.25** [0.16, 0.32]	0.40** [0.32, 0.47]	0.42** [0.34, 0.48]	—
Dedication	0.35** [0.28, 0.42]	0.37** [0.30, 0.42]	0.38** [0.31, 0.45]	—
Absorption	0.25** [0.17, 0.33]	0.31** [0.23, 0.38]	0.30** [0.22, 0.38]	—
Learning engagement	0.34** [0.26, 0.41]	0.43** [0.35, 0.49]	0.44** [0.36, 0.50]	—

a The Heterotrait-Monotrait (HTMT) values should be lower than 0.85 to indicate discriminant validity.

*p < 0.05; **p < 0.01; ***p < 0.001.

### Incremental validity

Finally, hierarchical regression was used to establish the incremental validity of the SOC. As shown in Table 6, the SOC subscales Selection, Optimization, and Compensation were included in each step of the sequential regression analysis. The statistically significant *R*^2^ change (Δ*R*^2^) in hierarchical linear regression indicated that the SOC subscales established incremental validity. However, the study also found that the association between SOC Selection and Vigor was positive but not statistically significant, with a negligible effect size (*β* = 0.003, *t* = 0.06).

**Table 6:** Regression analysis to test for the SOC on learning engagement.

	Variables	Vigor			Dedication			Absorption			Learning engagement		
		*β*	*t*	Δ*R*^2^	*β*	*t*	Δ*R*^2^	*β*	*t*	Δ*R*^2^	*β*	*t*	Δ*R*^2^
Step 1				0.01			0.02			0.01			0.01
	Age	0.03	0.54		−0.01	−0.2		0.06	0.99		0.03	0.59	
	Gender	−0.02	−0.43		0.07	1.77		0.01	0.32		0.03	0.68	
	Grade	−0.02	−0.41		0.03	0.48		−0.03	−0.53		−0.01	−0.21	
	Number of siblings	0.01	0.31		0.04	1.05		0.01	0.31		0.03	0.68	
	Household location	0.04	0.98		−0.01	−0.28		−0.06	−1.56		−0.02	−0.46	
	Family economic status (monthly income)	0.01	0.14		0.03	0.85		0.02	0.52		0.02	0.63	
Step 2				0.06***			0.11***			0.06***			0.11***
	Selection	0.003	0.06		0.17	3.66***		0.10	2.12*		0.11	2.45*	
Step 3				0.10***			0.05***			0.04***			0.09***
	Optimization	0.23	4.27***		0.16	3.10**		0.16	2.93**		0.22	4.22***	
Step 4				0.04***			0.02***			0.01*			0.03***
	Compensation	0.26	4.85***		0.19	3.44***		0.14	2.55*		0.23	4.39***	

### Reliability analysis

Four reliability measures—Cronbach’s *α*, McDonald’s omega, rho_a, and rho_c—were used to assess the internal consistency of the subscales. Table 7 summarizes the reliability coefficients of Cronbach’s *α*, rho_a, and rho_c. The Cronbach’s *α* coefficients for the three subscales all exceeded 0.70, indicating good reliability. Additionally, McDonald’s composite reliability (MCR) was calculated for each dimension. All three MCR values were greater than 0.60, demonstrating good composite reliability. All three rho_c coefficients exceeded 0.70, indicating significant subscale reliability. Similarly, the rho_a coefficients for the three subscales also showed acceptable reliability.

**Table 7:** The 17-item SOC’s 3-factor CFA results (*N* = 540).

Items	Standardized loading (*SE*)	Cronbach’s *α*	McDonald’s *ω*	rho_c	rho_a	Normed MSA	Cronbach’s alpha if item deleted	AVE
Selection								
S1	0.73 (0.04)***	0.88	0.88	0.88	0.88	0.96	0.93	0.55
S2	0.76 (0.03)***					0.95	0.93	
S3	0.74 (0.04)***					0.94	0.93	
S4	0.77 (0.03)***					0.94	0.93	
S5	0.75 (0.03)***					0.94	0.93	
S6	0.69 (0.03)***					0.95	0.93	
Optimization								
O1	0.83 (0.03)***	0.92	0.92	0.92	0.92	0.96	0.93	0.70
O2	0.83 (0.03)***					0.96	0.93	
O3	0.82 (0.03)***					0.96	0.93	
O4	0.82 (0.03)***					0.96	0.93	
O5	0.90 (0.02)***					0.92	0.93	
Compensation								
C1	0.65 (0.04)***	0.92	0.93	0.93	0.93	0.97	0.93	0.67
C2	0.87 (0.02)***					0.94	0.93	
C3	0.85 (0.03)***					0.96	0.93	
C4	0.82 (0.03)***					0.95	0.93	
C5	0.85 (0.03)***					0.96	0.93	
C6	0.86 (0.03)***					0.95	0.93	
*χ*^2^ = 182.11		RMSEA = 0.03			CFI = 0.99			
*χ*^2^/df = 1.57		90% CI = [0.02, 0.04]			GFI = 0.96			

## Discussion

The Selection, Optimization, and Compensation Questionnaire is a very standard tool that Freund and Baltes came up within 1998 to measure a person’s intentional self-regulation. Through the SOC Questionnaire, persons can manage resources and goals by such three strategies: selection, optimization, and compensation in facing various challenges. This paper deals with the factor structure, reliability, and validity of the Chinese version of the SOC Questionnaire among Chinese adolescents. In a sample of 1,080 Chinese high school students, EFA and CFA showed that the Chinese version is a highly reliable and valid tool to assess intentional self-regulation in adolescents using the SOC Questionnaire. The results of both EFA and CFA confirmed a three-factor measurement structure, as described, for selection, optimization, and compensation, similar to the findings by Freund and Baltes (1998) and later studies by Gestsdottir et al. (2010) and Farina and Johnson (2021). These results suggest that the Chinese version of the SOC Questionnaire not only shows consistency in theoretical factor structure but also is sound concerning reliability and validity tests. The instrument can, therefore, be effectively used for the assessment of intentional self-regulation among Chinese adolescents, which is an area highly relevant to intervention research in both education and mental health.

The Selection, Optimization and Compensation model provides a very comprehensive theoretical framework for goal-directed self-regulation through resourceful management and goals in the actualization of how individuals achieve this (Freund and Baltes, 2002; Geldhof et al., 2015a,b). The interaction among selection, optimization, and compensation strategies within SOC encourages positive development and general wellbeing in adolescents (Gestsdottir et al., 2015; Zając-Lamparska, 2021). It was, therefore, recommended that item 10 be removed from the SOC Questionnaire optimization subscale because it did not optimally discriminate amongst Chinese high school students, probably due to cultural differences between the East and West (McClelland and Wanless, 2015). Perseverance and striving are highly upheld in the Chinese spirit, encouraging one to persist and grasp the opportunity despite the challenges. In a cultural background in which resiliencies and persistence are major virtues, highly known and encouraged, students may instead focus more on the “important things” rather than “low chances of success,” hence leading to a cultural discrepancy in perceiving this item as compared to the West, which lays great emphasis upon rational decision-making and strategy adjustments. More than this, Western researchers might consider that people are supposed to quit when success is unlikely to optimize the allocation of resources (Li, 2012). In contrast, Chinese students further worked because their culture emphasizes perseverance. This led to a conceptual difference in how the measurement effects varied from one cultural context to another (Chen, 2012). These findings show the validity not just of the cross-cultural application of the SOC model but also if not more importantly, that understanding and respect toward cultural backgrounds are necessary for the research tools to be effective and reliable. Future research might want to discuss further how to apply the SOC Questionnaire in other cultures to tentatively understand and support adolescent development.

All the inter-factor correlations among these three subscales (Selection, Optimization, and Compensation) are positive and significant in the Chinese version of the SOC Questionnaire for Chinese adolescents. However, previous studies differed in strength. Compared with Millová and Malatincová’s study conducted in the Western cultural context in 2015, generally speaking, we find weaker correlations in S-O but more robust correlation coefficients in S-C for Chinese students. The relationship between Optimization and Compensation was also alterable in the Chinese sample. These differences may be due to cultural factors influencing intentional self-regulation (Gestsdottir et al., 2015; Millová and Malatincová, 2021). That is, individuals from the Chinese culture are more likely to utilize external resources and social support in attaining their goals, which would strengthen the O-C relation (Moghimi et al., 2021; Zając-Lamparska, 2021). According to research (Okabayashi, 2014), in Chinese culture, familial and educational expectations may outweigh personal choice and self-determination prevalent in Western cultures in selection strategies. For example, the relationship of selection to optimization strategies in Japanese culture also represents a trend similar to that obtained in Chinese, thus further underpinning the role of cultural differences in intentional self-regulation (Okabayashi, 2014). These cultural differences may have some critical implications for applying and interpreting the SOC Questionnaire. Further cross-cultural research might help better understand these observed differences and perhaps provide more empirical support to studies on intentional self-regulation across different cultural contexts (Gestsdottir et al., 2015; Schmitt et al., 2012). The more international comparative studies conducted, the closer we will be to more comprehensively revealing the application characteristics of the SOC Questionnaire in different cultural contexts, hence helping to further research in this area.

Within the step of estimating concurrent criterion validity, it is worth noting significant positive correlations of the three SOC Questionnaire subscales with learning engagement and its subscales, which provides evidence of the relation between intentional self-regulation and students’ learning engagement. The compensation strategy showed the most robust concurrent criterion validity with learning engagement. This indicates that it is one of the most critical strategies for enhancing students’ learning experiences (Coelho et al., 2019; Doo and Bonk, 2020). The selection and optimization strategies showed somewhat weaker concurrent criterion validity than did the compensation strategy. This is consistent with earlier studies conducted by other authors (Stefansson et al., 2018). The Conservation of Resources Theory tells that under stressful conditions, humans try to achieve, maintain, and protect resources (Hobfoll, 2011). Compensation strategies in learning contexts activate positive development based on using extrinsic resources (for example, help-seeking from peers or teachers) when encountering problems with one’s learning (Chauveron et al., 2016; Gestsdottir et al., 2015). Moreover, when all three SOC subscales entered together, compensation strategies correlated and predicted more highly than selection and optimization strategies. In other words, compensation strategies are central to the intentional self-regulation processes of students, more so in learning contexts. The SOC model underlines the interplay between selection, optimization, and compensation-based strategies that may have a high potential impact on adolescents about their learning engagement and intentional self-regulation accordingly (Stefansson et al., 2018). Therefore, this research touts the role of the Conservation of Resources Theory in enhancing learning engagements for adolescents. The role of compensation strategies as a core component of the SOC model is therefore underscored about theoretical understanding and practical application alike (Doo and Bonk, 2020; Gestsdottir et al., 2015). This thus suggests that there can never be any substitute for these in enhancing students’ learning engagement.

Although many studies have implemented SOC strategies all over the world, the literature has been insufficient for adolescents from other cultures (Geldhof et al., 2015a,b; Gestsdottir et al., 2015). This study fills the literature gap through the validation of the Chinese version of the SOC Questionnaire thus providing a reliable measurement tool in the assessment of intentional self-regulation for Chinese adolescents. Cross-cultural adaptation of the existing SOC Questionnaire is called for to consolidate cross-cultural differences in intentional self-regulation. According to (Millová and Malatincová, 2021; Moghimi et al., 2021), based on the results of the present study, the Chinese version of the SOC Questionnaire did very well in terms of factor structure, reliability, and validity, further validating its applicability among Chinese adolescents. In addition, it provides a reliable ground for further intervention research. It has identified SOC strategies that have found significant effects on enhancing the learning engagement of adolescents; this is, therefore an essential direction of interventions for the school and educational systems (Okabayashi, 2014; Schmitt et al., 2012). Like ability to support further the workability of the SOC Questionnaire in different school situations, it can also help the educator and the psychologist be more empirically supported and generate more targeted interventions aimed to improve the students’ coping with academic stress and mental health (Bowers et al., 2011; Guo et al., 2023; Zuo et al., 2024).

## Limitations and future research

Like any other research, the present study has a few limitations. First of all, this study was strongly oriented toward adolescents from general high schools who possessed higher academic expectations and better educational resources. The sample selection could undermine variations in the background and needs of students at vocational schools. The problems that vocational school students have are different and relate to lower academic expectations of them, access to educational resources, and social support. Future studies should administer the SOC Questionnaire in a sample of vocational school students to compare intentional self-regulation abilities among adolescents with different educational backgrounds. The current study further used a brief version of the SOC Questionnaire, which further enhanced the efficiency and practicality of the measurement for large-scale assessments in education and intervention studies (Gestsdottir et al., 2015). However, the analysis did not include other measures of intentional self-regulation for adolescents as standards. On this ground, future research should be equipped with multiple tools for measuring self-regulation so that there can be a more comprehensive validation of the SOC Questionnaire (Podsakoff et al., 2012). Third, the present study mainly relied on self-report measures, which are convenient and inexpensive but may result in social desirability and standard method bias (Podsakoff et al., 2003). In the future, research should include more diversified assessment methods like teacher reports, parent reports, and behavioral observations to obtain more heterogeneous data so that research findings are more solid. Finally, while in several cultural contexts, the SOC Questionnaire was shown to be applicable, there is still no “gold standard” for the construction of intentional self-regulation scales. Such future research should extend to a more rigorous validation of the SOC Questionnaire across different age stages and cultural contexts to change and develop intentional self-regulation over time (Gestsdottir et al., 2015).

## Conclusion

Results of the present study have shown that the Brief Version of the SOC Questionnaire is considerably reliable and a valid instrument within the Chinese context, therefore supporting its use within different national settings. This can be very useful for other researchers who may wish to conduct studies on intentional self-regulation among adolescents within the Chinese context.

## Data Availability

The datasets generated and/or analyzed during the current study are available from the corresponding author upon reasonable request.

## Footnotes

^1^ https://www.wjx.cn/
